# Down-regulated FTO and ALKBH5 co-operatively activates FOXO signaling through m6A methylation modification in HK2 mRNA mediated by IGF2BP2 to enhance glycolysis in colorectal cancer

**DOI:** 10.1186/s13578-023-01100-9

**Published:** 2023-08-14

**Authors:** Mujie Ye, Jinhao Chen, Feiyu Lu, Minghui Zhao, Suwen Wu, Chunhua Hu, Ping Yu, Jingbao Kan, Jianan Bai, Ye Tian, Qiyun Tang

**Affiliations:** 1 Department of Geriatric Gastroenterology, Institute of Neuroendocrine Tumor, Neuroendocrine Tumor Center, Jiangsu Province Hospital, The First Affiliated Hospital of Nanjing Medical University, Nanjing Medical University, NO.300 Guangzhou Road, Nanjing, 210029 China; 2 Department of Radiation Oncology, Jiangsu Province Hospital, The First Affiliated Hospital of Nanjing Medical University, Nanjing, China; 3 Obstetrics and Gynaecology Hospital, Fudan University, Shanghai, China

**Keywords:** Colorectal cancer, N6-methyladenosine, RNA demethylase, Glycolysis

## Abstract

**Background:**

N6-methyladenosine (m6A) modification is the most abundant reversible methylation modification in eukaryotes, and it is reportedly closely associated with a variety of cancers progression, including colorectal cancer (CRC). This study showed that activated lipid metabolism and glycolysis play vital roles in the occurrence and development of CRC. However, only a few studies have reported the biological mechanisms underlying this connection.

**Methods:**

Protein and mRNA levels of FTO and ALKBH5 were measured using western blot and qRT-PCR. The effects of FTO and ALKBH5 on cell proliferation were examined using CCK-8, colony formation, and EdU assays, and the effects on cell migration and invasion were tested using a transwell assay. m6A RNA immunoprecipitation (MeRIP) and RNA-seq was used to explore downstream target gene. RIP was performed to verify the interaction between m6A and HK2. The function of FTO and ALKBH5 in vivo was determined by xenograft in nude mice.

**Results:**

In this study, FTO and ALKBH5 were significantly down-regulated in CRC patients and cells both in vivo and in vitro in a high-fat environment. Moreover, FTO and ALKBH5 over-expression hampered cell proliferation both in vitro and in vivo. Conversely, FTO and ALKBH5 knockdown accelerated the malignant biological behaviors of CRC cells. The mechanism of action of FTO and ALKBH5 involves joint regulation of HK2, a key enzyme in glycolysis, which was identified by RNA sequencing and MeRIP-seq. Furthermore, reduced expression of FTO and ALKBH5 jointly activated the FOXO signaling pathway, which led to enhanced proliferation ability in CRC cells. IGF2BP2, as a m6A reader, positively regulated HK2 mRNA in m6A dependent manner. Additionally, down-regulation of FTO/ALKBH5 increased METTL3 and decreased METTL14 levels, further promoting CRC progression.

**Conclusion:**

In conclusion, our study revealed the FTO-ALKBH5/IGF2BP2/HK2/FOXO1 axis as a mechanism of aberrant m6A modification and glycolysis regulation in CRC.

**Supplementary Information:**

The online version contains supplementary material available at 10.1186/s13578-023-01100-9.

## Background

Colorectal cancer (CRC) is one of the most commonly diagnosed malignancies worldwide [1]. The prognosis of CRC patients is not very desirable as most patients are diagnosed at an advanced stage due to a lack of early symptoms. The existing treatment regimen is limited to surgical excision and chemotherapy [2]. Therefore, established biomarkers are urgently required for early diagnosis and novel targeted therapeutics for patients with CRC.

N6-methyladenosine (m6A) is the most pervasive internal modification form of eukaryotic mRNA and is implicated in gene dysregulation through the methylation of the adenosine base at the nitrogen-6 position [3]. m6A-mediated mRNA modification is associated with multiple biological processes including stem cell differentiation and self-renewal, DNA damage response, and tumor development [4, 5]. The m6A-dependent modification is reversible and regulated by methyltransferases (METTL3, METTL14, and WTAP), demethylases (FTO and ALKBH5), and m6A-binding proteins (such as YTH domain family proteins and IGF2BP family proteins), which are essential for cancer initiation and progression [6].

Demethylases such as obesity-associated protein (FTO) and alkylation repair homolog protein 5 (ALKBH5) regulate methylation reversal, whereby their implications in carcinogenesis have been reported in multiple cancers [7]. Hu et al. showed that up-regulation of FTO activated HSF1 and promoted the progression of multiple myeloma [8]. Moreover, ALKBH5 was reported to regulate the expression of USP22 and RNF40, enhancing the growth of osteosarcoma [9]. Accordingly, the roles of FTO and ALKBH5 in CRC warrant further investigation.

Common features of cancer include high levels of glycolysis and lactic acid formation under aerobic conditions. The accumulation of glucose in tumor tissue provides a massive source of energy for tumor progression [10]. Glycolysis-related genes and transcriptional regulators have been found to correlate with the poor prognosis of various malignant tumors [11]. More interestingly, m6A modification is involved in regulating glycolysis in CRC [12], however, its role in glycolysis modification remains unclear. Hexokinase 2 (HK2) is an enzyme that phosphorylates glucose to produce glucose-6-phosphate and regulates the first committed step in glucose metabolism [13, 14]. HK2 is highly expressed in embryonic tissues, however, its expression terminates after growth and becomes limited to very few tissues. However, HK2 expression sharply increases after normal cells are converted to cancer cells, which is considered a hallmark of many malignant tumors [15, 16]. HK2 presents another perspective on the role of glucose metabolism in tumor progression, however, the underlying mechanisms still need to be determined.

In this study, we discovered that the demethylases FTO and ALKBH5 were down-regulated in CRC, especially in obese patients. Functional assays indicated an anti-cancer role of FTO and ALKBH5 in CRC. The mechanism of action of FTO and ALKBH5 involved the glycolysis via regulation of HK2 expression in an m6A-dependent manner by IGF2BP2. Moreover, FTO and ALKBH5 negatively regulated METTL3 and positively regulated METTL14 expression levels, enhancing their role as tumor suppressors of CRC.

## Materials and methods

### Specimens and cell lines

Thirty-six pairs of frozen tissues were obtained from patients diagnosed with CRC who also underwent surgical excision at the Jiangsu Province Hospital. All protocols were reviewed and approved by the Ethics Committee of Nanjing Medical University. SW620 and HCT116 cell lines were purchased from the Chinese Academy of Science (Shanghai, China) and respectively cultured in a Leibovitz’s L-15 medium (Fuheng, Shanghai, China) and RPMI-1640 medium (Biological Industries, Israel). DLD1, RKO and NCM460 cells were gifts from Changhong Miao of Fudan University Shanghai Cancer Center. Cell culture mediums were supplemented with 10% fetal bovine serum (FBS; Yeasen, Shanghai, China) and 1% penicillin–streptomycin (Yeasen) in a humidified incubator with 5% CO_2_ at 37 °C.

### Plasmid transfection

ALKBH5, FTO, METTL3, METTL14, WTAP, HK2 and IGF2BPs knockdowns in addition to over-expression plasmids were synthesized by Shanghai Genomeditech Biotech Co. Ltd. 293 T cells were used for lentivirus packaging with PEIMAX treatment (Polysciences, USA). After infection with a concentrated virus and 5 μg/ml polybrene (Genomeditech) for 48 h, stably transfected cells were screened through treatment with 5 μg/mL puromycin (Yeasen). Western blotting was performed to verify the efficiency of transfection efficiency. All shRNA targets were listed in Additional file 6: Table S1.

### Cell proliferation

For the cell counting kit 8 (CCK-8) assay, 5 × 10^3^ CRC cells were seeded in 96-well plates and counted 72 h in the respective media with 10% FBS. Cell proliferation was measured at 450 nm absorbance by adding 10 μL in CCK-8 (Yeasen) after 2 h. For the colony formation assay, 1 × 10^4^ cells were seeded in 6-well plates and cultured in respective media with 10% FBS for 7 days. Images were then captured after the cells were fixed with 4% formaldehyde for 30 min and stained with 2% crystal violet (Yeasen) for another 30 min. For the EdU assay, cells seeded in 96-well plates were cultured with 50 μM EDU for 2 h and then fixed with 4% paraformaldehyde. The cells were then incubated with 1 × Apollo reaction cocktail (RiboBo, Guangzhou, China) for 30 min after permeabilization with 0.5% Triton-X. After DNA staining with Hoechst33342, the images were finally visualized using a fluorescence microscope (Zeiss, Germany).

### Cell migration and invasion assay

Cells were suspended in a medium without FBS and seeded in 8 μm pore inserts (Corning, USA) at a density of 2 × 10^4^ cells per insert. The inserts were placed into 24-well plates containing 800 μL of 30% FBS medium. After culturing for 48 h, the cells on the upper side of the filter were fixed with 4% formaldehyde for 30 min and stained with 2% crystal violet for another 30 min. The cells on the upper side were then wiped with swabs, while the cells on the lower side were imaged randomly under a microscope.

### Quantitative Real-Time PCR (qRT-PCR)

Total RNA was extracted from cells using TRIzol reagent (Invitrogen, USA), and cDNA was synthesized using 4xHifair^®^ III SuperMix plus (Yeasen) according to the manufacturer’s protocols. qRT-PCR was finally performed with a Roche machine using SYBR Green PCR master mix (Yeasen). GAPDH was used as the internal reference. The qPCR primers used in this study are listed in Additional file 6: Table S2.

### Western blotting

Cells were lysed in NP40 buffer containing protease and phenylmethanesulfonyl fluoride (PMSF, 2 mM) for 30 min and then boiled with 1 × loading buffer at 105 °C for 10 min. The extracted proteins were separated using sodium dodecyl sulfate–polyacrylamide gel (SDS-PAGE) electrophoresis and transferred onto a nitrocellulose filter membrane. The membranes were blocked using 8% nonfat milk for 2 h and then washed three times with Tris-Buffered Saline Tween-20 buffer. The blocked membranes were incubated with primary antibodies at 4 ℃ overnight, followed by respective secondary antibodies (1:5000) at room temperature for 2 h. Antibody information is listed in Additional file 6: Table S3. The signals were developed using the image lab software with an enhanced chemiluminescence reagent kit (New Cell & Molecular Biotech, Suzhou, China).

### m6A RNA methylation quantification

N6-methyladenosine RNA methylation was quantified using an m6A RNA methylation quantification kit. Briefly, 300 ng of RNA were bound to wells and then incubated with capture antibody for 1 h. The mixture was then incubated with detection antibodies for 30 min and reacted with an enhancer solution for another 30 min at room temperature, the reaction signal was read using a microplate spectrophotometer at 450 nm.

### RNA immunoprecipitation (RIP) assays

Cells were lysed with RIP lysis buffer and then incubated with specific antibodies at 4 ℃ for 2 h. The mixture was then incubated with protein A/G magnetic beads at 4 °C overnight. The bound RNA was separated from the beads by washing with RIP buffer solution. The eluted RNA was finally extracted and reverse-transcribed into cDNA for further analysis using qRT-PCR.

### Immunohistochemical analysis

Human CRC and paired adjacent normal tissues were soaked in paraffin and fixed with 4% formaldehyde. After cutting and dewaxing, the slices were incubated in citric acid antigen retrieval buffer and blocked with 3% BSA blocking buffer, followed by incubation with primary antibodies at 4 ℃ overnight. Next, slices were incubated with secondary antibodies for 1 h at room temperature after being washed three times. The DAB color-developing solution was added and hematoxylin staining was performed. Finally, the slices were dehydrated and images were taken randomly by scanning.

### Glucose and ATP level detection

For glucose assay, the cell supernatant was collected, 20ul of the sample to be tested was added to each well, and 100ul of the working solution of the substrate was added and incubated at 37 ℃ for 5 min to read the OD value A1. Then each well was added with enzymic antibody working solution of 20ul, and the reaction plate was placed at 37 ℃ for 15 min after shaking and mixing. The absorption value A2 was measured at 550 nm with enzymic marker within 30 min. All OD values are calculated by subtracting A1 from A2. For ATP level assay, the culture solution was removed, and the lysate was added in the ratio of 200 µl to each well of the 6-well plate. The lysate was blown repeatedly to fully lysate the cells. After lysate, the supernatant was taken for subsequent determination after centrifugation at 12000 g at 4 ℃ for 5 min. Then, 100 µl of ATP detection solution was added to each well and placed it at room temperature for 3–5 min, so that all the background ATP is consumed, thus reducing the background. A sample of 20 µl was then added to the test hole and the value was measured with a chemiluminescence instrument.

### Animal experiments

For the mouse xenograft model, 2 × 10^6^ cells were injected subcutaneously into the flank regions of female BALB/c nude mice (4–5 weeks). The animal procedures were approved by the Animal Care and Use Committee of Nanjing Medical University. Five weeks after injection, the tumors were excised from the mice and the weight, width (W), and length (L) were measured. The volume (V) of each tumor was calculated using the formula: V = (W^2^ × L/2).

### Statistical analysis

All experiments were independently repeated at least three times. The means between groups were compared using unpaired or paired student’s t-tests. All data were presented in the form of mean ± SD and p < 0.05 was considered statistically significant.

## Results

### m6A demethylase FTO and ALKBH5 expression is down-regulated in CRC

**Fig. 1:**
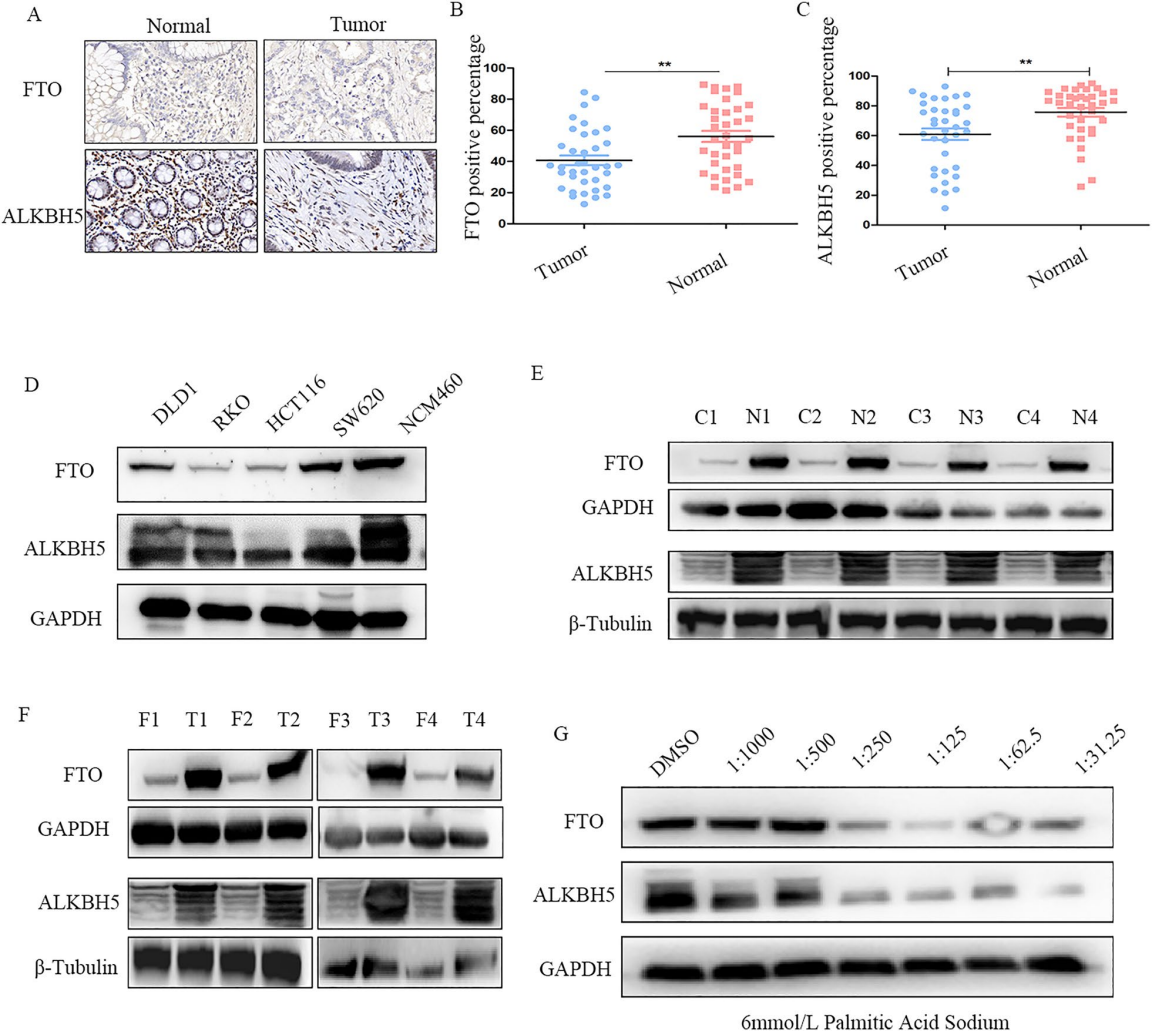
The expression of FTO and ALKBH5 in human colorectal cancer. **A** Representative images of IHC staining *FTO and ALKBH5* in TMAs. **B**, **C** The positive percentage of FTO and ALKBH5 in CRC tissues and normal tissues. **D** Western blot analysis of FTO and ALKBH5 expression in CRC cancer cells and NCM460 cells. **E** The expression of FTO and ALKBH5 in representative CRC tissues and adjacent normal tissues was indicated by western blot. **F** The expression of FTO and ALKBH5 in obesity CRC group and normal weight CRC groups. **G** Western blot showing FTO and ALKBH5 expression in CRC cells treated with palmitic acid in different concentrations for 24 h. **p < 0.01

To evaluate the expression of FTO and ALKBH5 in human CRC samples, immunohistochemistry (IHC) staining was used to visualize the results of the tissue microarrays (TMAs) that contained 36 samples (Fig. 1A). The positive percentages of FTO and ALKBH5 were significantly lower in CRC tissues than in normal tissues (Fig. 1B, C). The protein expression was also analyzed showing low expression levels of FTO and ALKBH5 in several CRC cell lines than that in normal epithelial cell lines NCM460 (Fig. 1D). Similarly, four representative CRC tissues showed lower FTO and ALKBH5 expression levels than the corresponding para-cancer tissues (Fig. 1E). As FTO is known as an obesity-associated protein, we aim to explore whether obese CRC patients are more susceptible to suffering from CRC through dynamic m6A changes. Interestingly, the protein expression levels of FTO and ALKBH5 were lower in the obese CRC patients (Fig. 1F). A high-fat environment was also achieved using a palmitic acid-treated medium instead of a normal medium. The results revealed that FTO and ALKBH5 expression levels significantly decreased with an increase in the concentration of palmitic acid (Fig. 1G).

### FTO inhibits cell proliferation, migration, and invasion in vitro

**Fig. 2:**
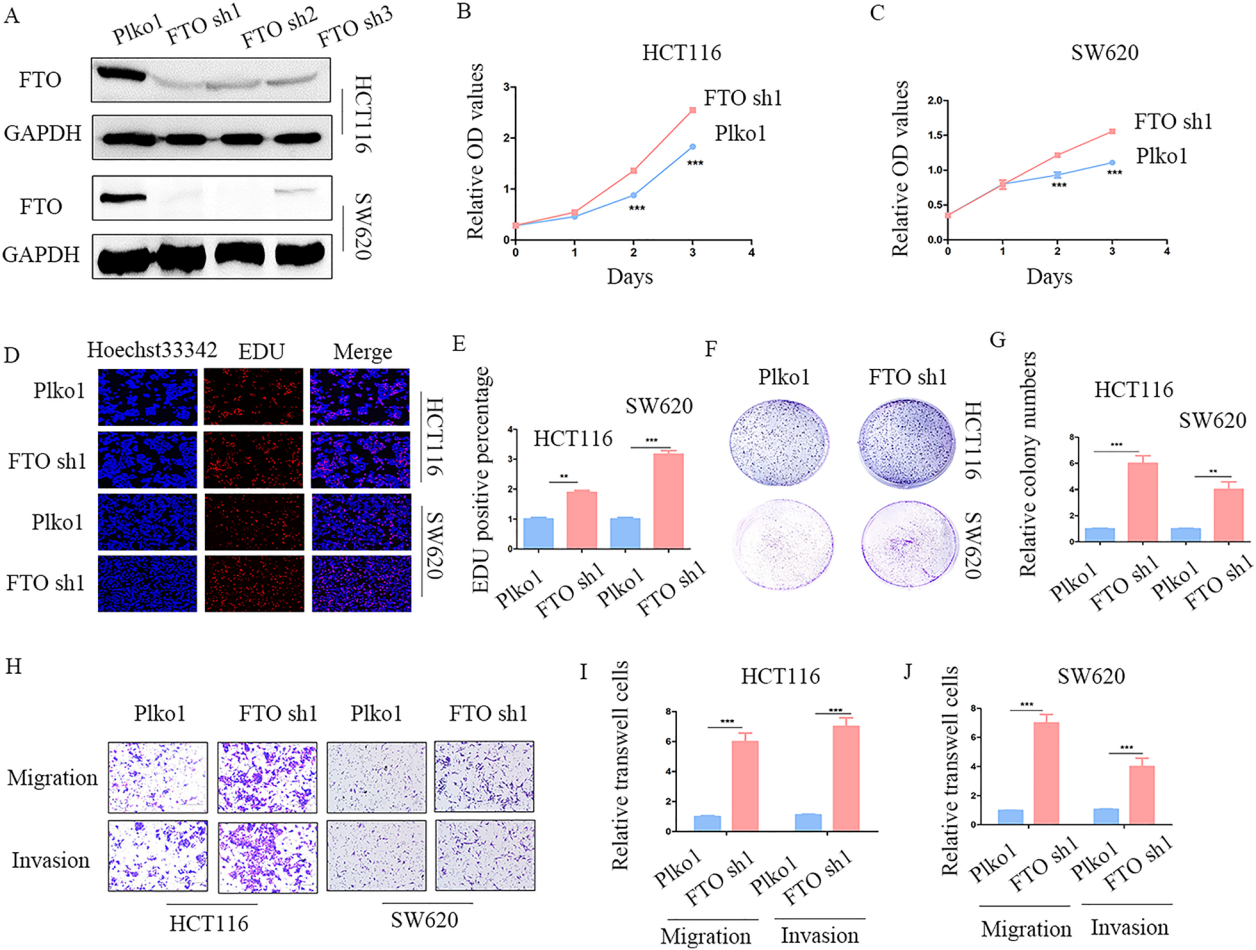
Knockdown of FTO promotes proliferation, migration, and invasion of CRC **A** Knockdown of FTO by three different shRNAs in two CRC cell lines verified by western blot. **B**–**G** The results of CCK-8, colony formation, and EdU assays in FTO knockdown stably cell lines and control groups, together with its statistical chart (magnification times, 200 ×). **H**–**J** Transwell results of FTO knockdown in HCT116 and SW620 cells, together with its statistical chart (magnification times, 100 ×). **p < 0.01,***p < 0.001

To examine the effect of FTO expression on CRC growth, stably transfected cell lines with FTO knockdown and over-expression were constructed using lentiviral transfection technology. The efficiency of FTO knockdown and over-expression was verified by western blotting (Fig. 2A, Additional file 1: Figure S1A). Subsequently, CCK-8 assays were performed, which showed that FTO knockdown significantly promoted proliferation in CRC cells while FTO over-expression inhibited their proliferation (Fig. 2B, C, Additional file 1: Figure S1B, C). The results of EdU assays also demonstrated that FTO deficiency remarkably expedited the growth of CRC cells, whereas FTO up-regulation restricted the proliferation of CRC cells (Fig. 2D, E, Additional file 1: Figure S1D, E). Similar findings were obtained using the colony formation assay (Fig. 2F, G, Additional file 1: Figure S1F, G). Additionally, FTO knockdown promoted the migration and invasion of CRC cells, as indicated by the transwell assays (Fig. 2H–J, Additional file 1: Figure S1H–J). Therefore, the above results demonstrate that the increased expression of FTO in CRC cells suppresses cell proliferation, migration, and invasion in vitro.

### ALKBH5 restrains cell proliferation, migration, and invasion in vitro

**Fig. 3:**
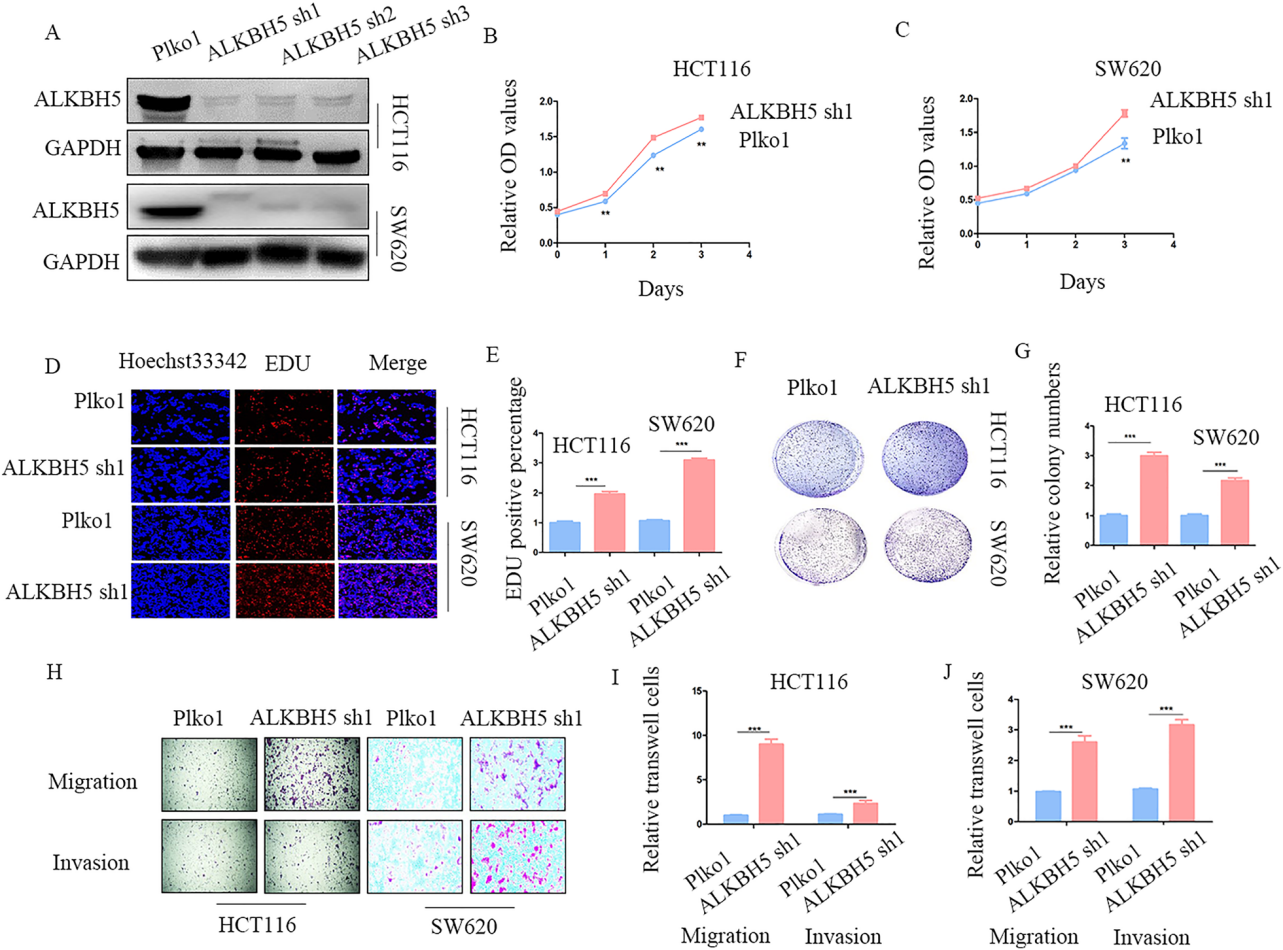
ALKBH5 knockdown accelerates proliferation, migration, and invasion of CRC **A** The knockdown efficiency of ALKBH5 showed by western blot. **B**, **C** CCK-8 analysis of the effects of ALKBH5 knockdown on the CRC cell viability. **D**, **E** EdU analysis of the effects of ALKBH5 knockdown on the proliferation ability (magnification times, 200 ×). **F**, **G** Colony formation analysis of ALKBH5 knockdown on the colony formation ability. **H**–**J** Transwell of ALKBH5 knockdown on the CRC cell migration and invasion (magnification times, 100 ×). **p < 0.01; ***p < 0.001

To explore the role of ALKBH5 expression in CRC, we established a stable ALKBH5 knockdown and over-expression model in both HCT116 and SW620 cell lines using specific shRNAs and found that the protein levels of ALKBH5 were significantly inhibited and over-expressed respectively (Fig. 3A, Additional file 2: Figure S2A). The cell growth curve indicated that ALKBH5 knockdown accelerated cell proliferation in two CRC cell lines, however, ALKBH5 over-expression showed the opposite results (Fig. 3B, C, Additional file 2: Figure S2B, C). Similarly, the EdU assays showed an anti-proliferative role of ALKBH5 in CRC cell lines (Fig. 3D, E, Additional file 2: Figure S2D, E). Colony formation experiments revealed that ALKBH5 inhibition increased the number of colonies, whereas ALKBH5 over-expression suppressed colony formation (Fig. 3F, G, Additional file 2: Figure S2F, G). Additionally, the results from the transwell assay showed that ALKBH5 knockdown significantly facilitated cell migration and invasion of CRC cells, whereas, up-regulation had the opposite effect (Fig. 3H–J, Additional file 2: Figure S2H–J). In conclusion, these results show that ALKBH5 can hinder cell proliferation, migration, and invasion of CRC cells.

### FTO and ALKBH5 inhibits FOXO signaling pathway via negatively regulation of HK2

**Fig. 4:**
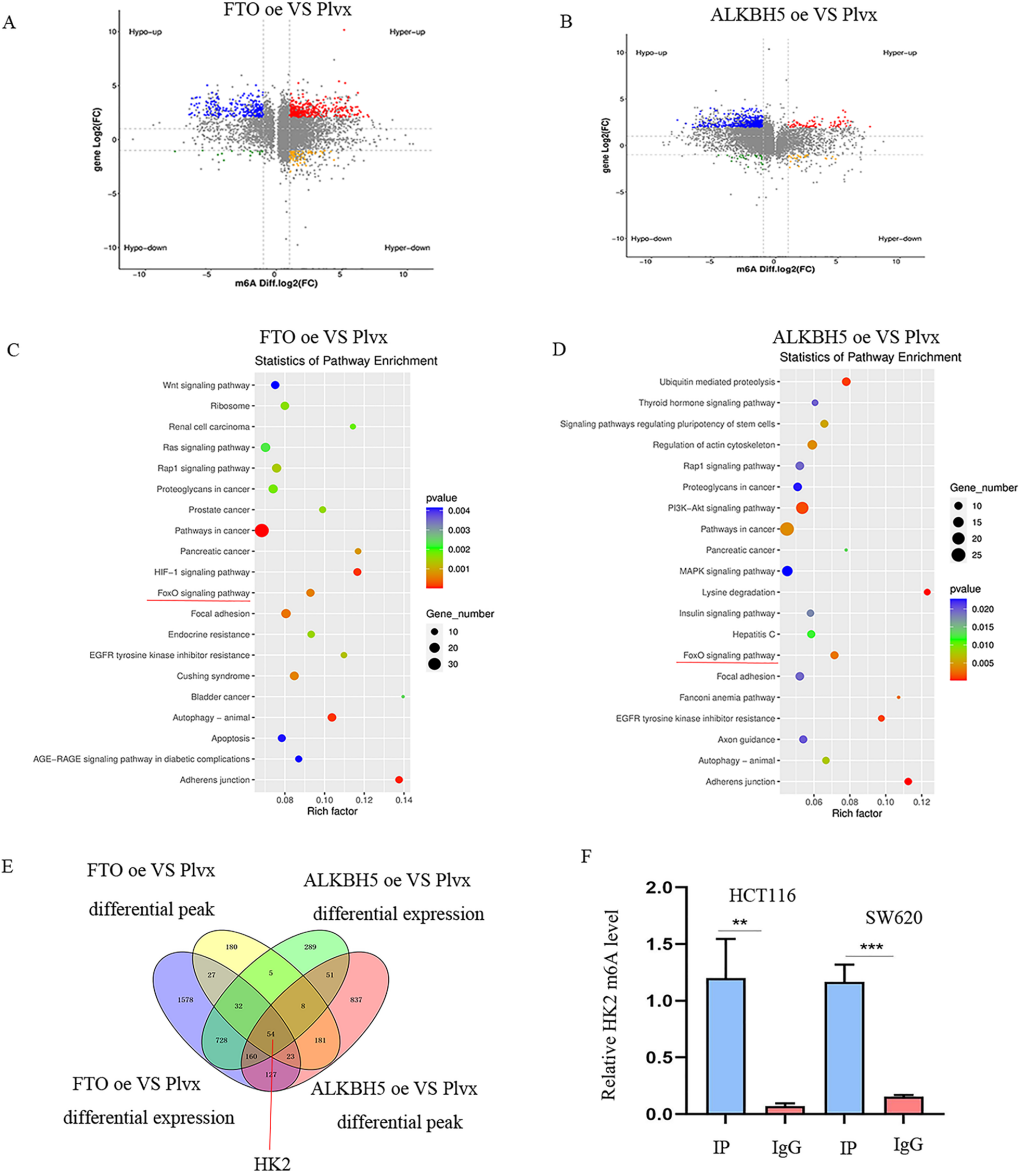
FTO and ALKBH5 restrains tumor progression via regulating HK2. **A**, **B** Volcano plots of the differential mRNA levels and m6A modification levels of FTO over-expression and ALKBH5 over-expression compared to Plvx groups by RNA-seq and MeRIP-seq. **C**, **D** The KEGG pathway enrichment in FTO over-expression and ALKBH5 over-expression is indicated by a bubble plot. **E** Venn diagram showed the overlap genes identified by RNA-seq and MeRIP-seq analysis. **F** The levels of HK2 mRNA were examined by RIP RT-PCR in HCT116 cells and SW620 cells. **p < 0.01; ***p < 0.001

To investigate the underlying molecular mechanisms of FTO and ALKBH5 in CRC progression, we conducted RNA sequencing (RNA-seq) and m6A-modified RNA immunoprecipitation sequencing (MeRIP-seq) in CRC cells with stable FTO over-expression, ALKBH5 over-expression, and control cells. The differential mRNA and differential m6A peaks modified genes are shown in volcano plots (Fig. 4A, B). Through KEGG pathway enrichment analysis, FOXO signaling was identified as a common pathway (Fig. 4C, D). Furthermore, the common target gene HK2, regulated by FTO and ALKBH5, was identified through the intersection between RNA-seq and MeRIP-seq (Fig. 4E). Therefore, we verified whether HK2 is regulated by FTO and ALKBH5 in an m6A-dependent manner. m6A IP was conducted, which demonstrated the successful interaction between m6A and HK2 mRNA (Fig. 4F). Moreover, knockdown of FTO/ALKBH5 increased HK2 m6A modification, while over-expression them decreased this modifications (Additional file 5: Figure S5A).

### FTO and ALKBH5 diminishes CRC malignant progression by inactivating FOXO signaling pathway mediated by HK2

**Fig. 5:**
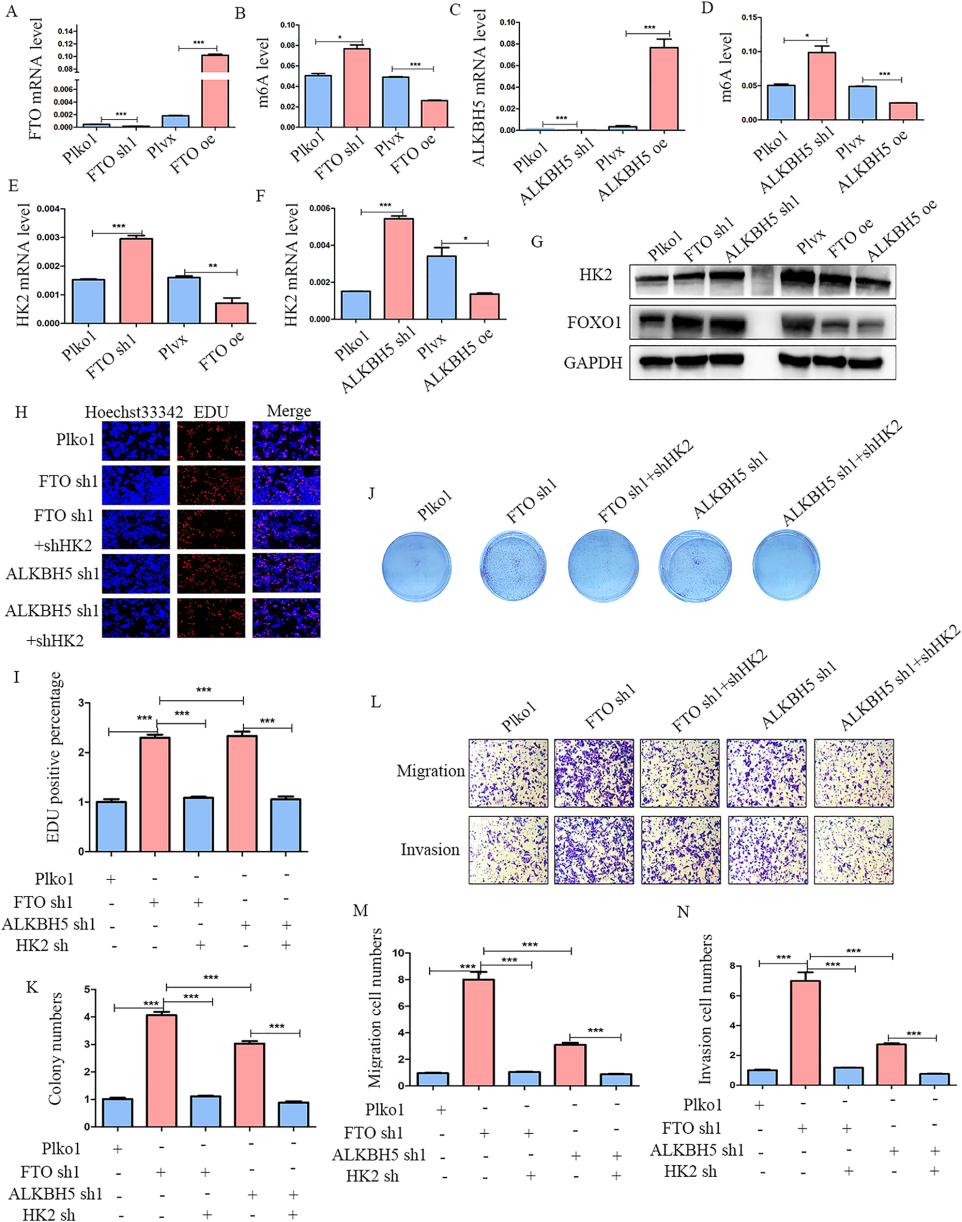
Down-regulation of FTO and ALKBH5 facilitates CRC progression via activating FOXO1 mediated by HK2 **A**, **B** The mRNA and m6A levels of FTO knockdown and over-expression in HCT116 cells. **C**, **D** The mRNA and m6A levels of ALKBH5 knockdown and over-expression in HCT116 cells. **E**, **F** The HK2 expression was measured by RT-PCR in FTO knockdown/over-expression, and ALKBH5 knockdown/over-expression in HCT116 cells was verified by qPCR. **G** Western blot showing HK2 and FOXO1 expression in CRC cells treated with FTO knockdown/over-expression, ALKBH5 knockdown/over-expression in HCT116. **H**–**K** CCK-8, colony formation, and EdU assays all indicated the ability of proliferation in FTO/ALKBH5 deficient CRC cells was rescued by HK2 knockdown. **L**–**N** Analysis of CRC cell migration and invasion while transfected with shHK2 in FTO/ALKBH5 deficient CRC cells.*p < 0.05; **p < 0.01; ***p < 0.001

The stable cell lines of FTO and ALKBH5 knockdown and over-expression were verified by RT-PCR (Fig. 5A, C). Moreover, we checked the m6A level after the up/down-regulation of FTO/ALKBH5. As expected, down-regulating FTO or ALKBH5 increased, while up-regulation decreased the total m6A level (Fig. 5B, D). The mRNA levels of HK2 also indicated a negative relationship between FTO and ALKBH5 (Fig. 5E, F). Moreover, the protein levels of HK2 were significantly increased when FTO and ALKBH5 were depleted, whereas over-expression of FTO and ALKBH5 inhibited HK2 protein levels (Fig. 5G). To verify the activation of the FOXO signaling pathway medicated by down-regulation of FTO and ALKBH5 in CRC cells, we analyzed the protein expression of FOXO1 through western blotting. These results showed that the FOXO signaling pathway was activated in FTO and ALKBH5 knockdown cell lines, whereas up-regulation of FTO and ALKBH5 inhibited the FOXO signaling pathway (Fig. 5G).

Additionally, rescue experiments further validated the functional relationship between HK2 and FTO/ALKBH5. We performed EdU and colony formation assays and found that FTO/ALKBH5 knockdown accelerated the proliferation of CRC cells, whereas HK2 knockdown in FTO/ALKBH5 stable knockdown CRC cells decreased cell proliferation (Fig. 5H–K). Additionally, we validated the effects of migration and invasion for HK2 in FTO and ALKBH5 stable knockdown CRC cells. The results showed that silencing HK2 led to the attenuation of cell migration and invasion induced by FTO/ALKBH5 knockdown (Fig. 5L–N). In addition to knocking down HK2, we also utilized hexokinase inhibitor, 2-Deoxy-D-glucose (2DG), to treat HCT116 cells. Similarly, 2DG restored the FOXO pathway activation (Additional file 3: Figure S3A) and increased cell proliferation (Additional file 3: Figure S3B), colony formation (Additional file 3: Figure S3C, D), migration and invasion (Additional file 3: Figure S3E–G) induced by FTO and ALKBH5 silence. Furthermore, treating HCT116 cells with 2DG alone also inhibited cell proliferation (Additional file 4: Figure S4B), colony formation (Additional file 4: Figure S4C–D), migration and invasion (Additional file 4: Figure S4E–F) by inactivating FOXO pathway (Additional file 4: Figure S4A). Overall, these results indicates that a deficiency in FTO and ALKBH5 functions as oncogenes via the promotion of FOXO1 mediated by HK2 in CRC.

### FTO and ALKBH5 abrogates tumor growth in vivo

**Fig. 6:**
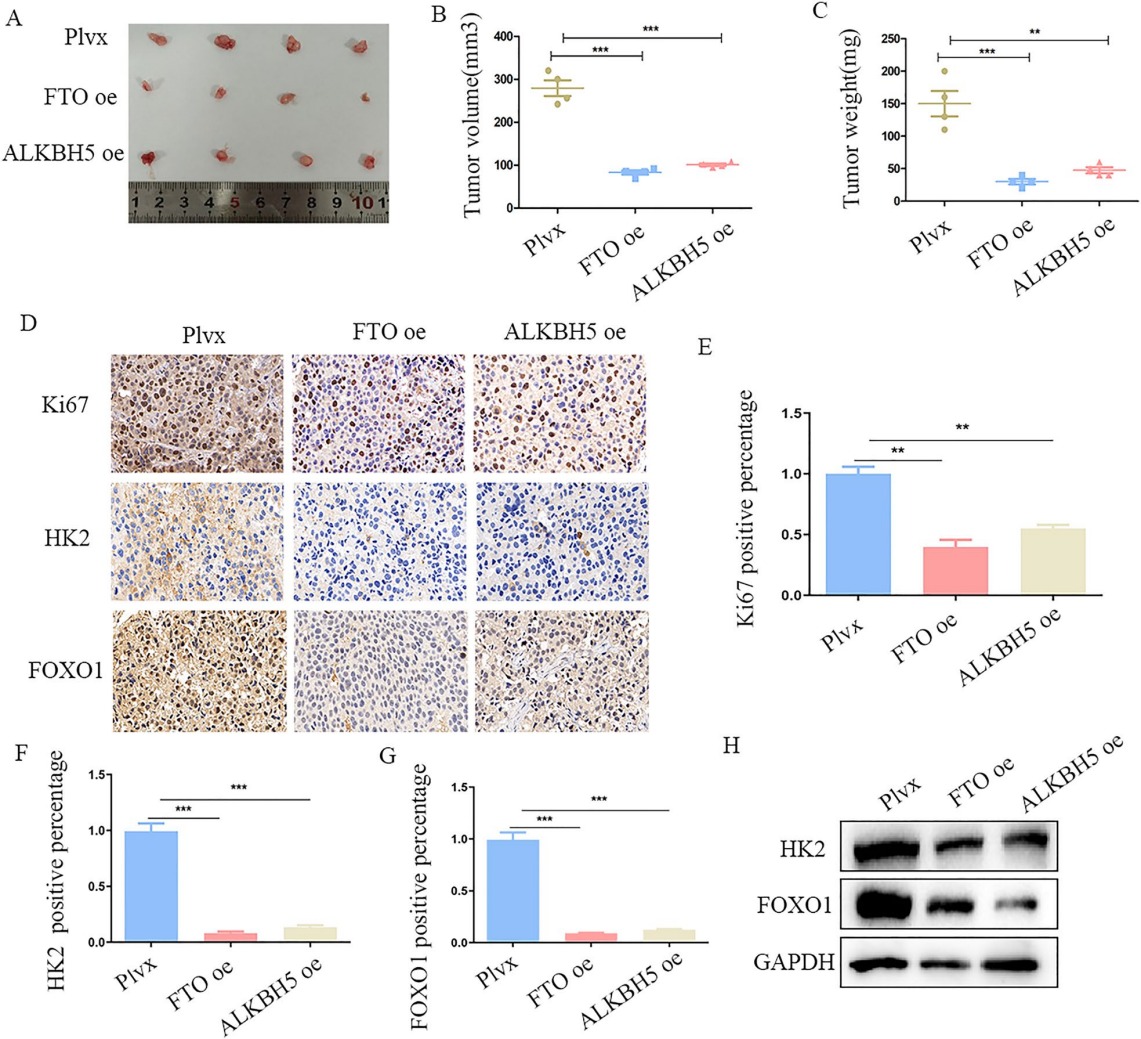
FTO and ALKBH5 inhibits tumor growth in vivo. **A** Image of subcutaneous implantation tumors from FTO over-expression and ALKBH5 over-expression compared to Plvx groups. **B**, **C** Tumors were removed 4 weeks after subcutaneous implantation, followed by volume calculation and weight measurement. **D**–**G** Representative IHC staining images of Ki67/HK2/FOXO1 and quantification of Ki67/HK2/FOXO1 positive rate. **H** Western blot for HK2 and FOXO1 in FTO over-expression and ALKBH5 over-expression compared to Plvx groups in tumors. **p < 0.01; ***p < 0.001

To confirm the bio-function of FTO and ALKBH5 in vivo, we subcutaneously injected CRC cells over-expressing FTO and ALKBH5 as well as an empty vector into nude mice. The groups with over-expressed FTO and ALKBH5 showed a significant decrease in tumor growth, both in terms of tumor weight and volume, compared with those of the control group (Fig. 6A–C). Further IHC and western blot assay indicated that up-regulation of FTO and ALKBH5 decreased the level of the proliferation marker Ki67 level, as well as HK2 and FOXO1 in vivo(Fig. 6D–H). As HK2 is a rate-limiting enzymes of the glycolytic pathway, we also explore the effect of FTO and ALKBH5 on glucose metabolism. Results showed down-regulation of FTO and ALKBH5 consumed more glucose and produced more ATP (Additional file 5: Figure S5B, C). To study whether FTO/ALKBH5 has an effect on apoptosis, we performed flow cytometry, tunel staining and western blot for Bcl2 and Caspase3, however, it showed no significant difference with FTO/ALKBH5 over-expression (Additional file 5: Figure S5D–F). Together, these results demonstrate FTO and ALKBH5 inhibits tumor growth in vivo and may participates glucose metabolism.

### FTO and ALKBH5 regulates HK2 in m6A-IGF2BP2 dependent manner

**Fig. 7:**
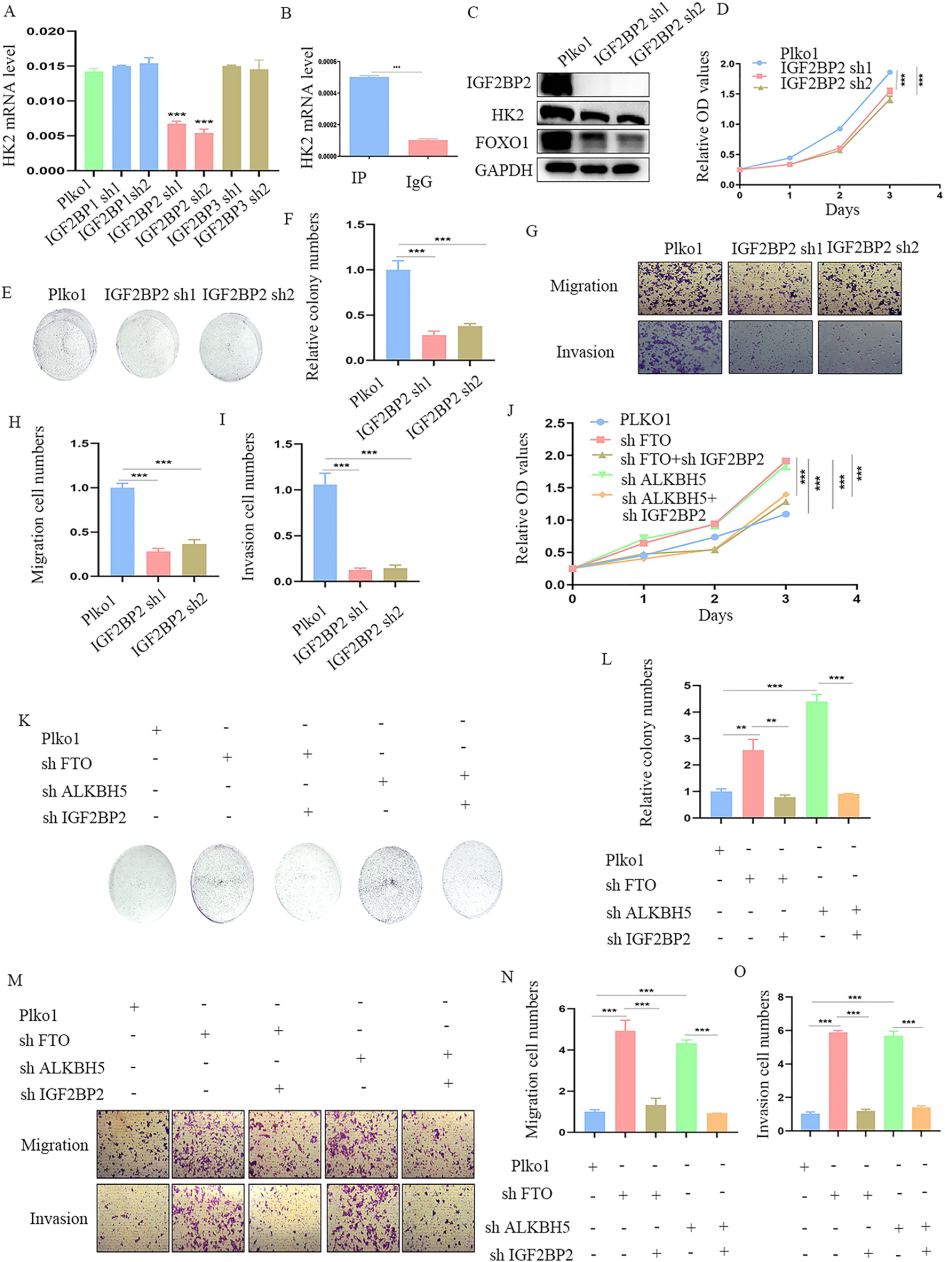
FTO and ALKBH5 regulates HK2 dependent on IGF2BP2. **A** HK2 mRNA was detected by QPCR after IGF2BP1/2/3 knockdown. **B** RIP-QPCR was performed by IGF2BP2 protein and HK2 mRNA. **C** IGF2BP2, HK2 and FOXO1 protein levels were detected by western blot in IGF2BP2 knockdown cells. **D**–**F** CCK8 and colony formation assays were performed in IGF2BP2 down-regulation cells. **G**–**I** Cell migration and invasion was analysed in IGF2BP2 deficient HCT116 cells. **J**–**L** Both CCK-8 and colony formation assays indicated the ability of proliferation in FTO/ALKBH5 silence HCT116 cells was restored by IGF2BP2 knockdown. **M**–**O** Analysis of cell migration and invasion while transfected with shIGF2BP2 in FTO/ALKBH5 deficient HCT116 cells (magnification times, 100 ×). **p < 0.01; ***p < 0.001

Since HK2 was positively regulated by m6A methylation, we further explore which m6A reader participates in m6A methylation of HK2 mRNA. So, we searched in RM2Target database(http://rm2target.canceromics.org) and found IGF2BP family proteins may affect the m6A modification of HK2. To validate our hypothesis, we silenced IGF2BP1, IGF2BP2, and IGF2BP3 respectively in HCT116 cells, and QPCR indicated that only IGF2BP2 down-regulation led to HK2 decrease (Fig. 7A). Then, RIP-QPCR was performed using anti-IGF2BP2 to confirm the interaction between HK2 mRNA and IGF2BP2 in CRC cells. Notably, IGF2BP2 also bound to the mRNA of HK2 in CRC cells (Fig. 7B). Next, we performed western blot and the results showed both HK2 and FOXO1 were inhibited by IGF2BP2 knockdown (Fig. 7C). Moreover, cell functional assay demonstrated IGF2BP2 silence significantly decreased cell proliferation (Fig. 7D), colony formation (Fig. 7E–F) as well as cell migration and invasion of CRC cells (Fig. 7G–I). In addition, we also knocked down IGF2BP2 in FTO and ALKBH5 down-regulation stabled-transfected cells. The results indicated that IGF2BP2 silencing decreased the malignant behaviors of CRC cells in proliferation (Fig. 7J), colony formation (Fig. 7K–L), migration, and invasion (Fig. 7M–O) induced by FTO and ALKBH5 silence. In summary, FTO and ALKBH5 regulates HK2 in m6A-IGF2BP2 dependent manner.

### Silenced FTO or ALKBH5 increases METTL3 while decreasing METTL14 to promote CRC malignant biological behaviors

**Fig. 8:**
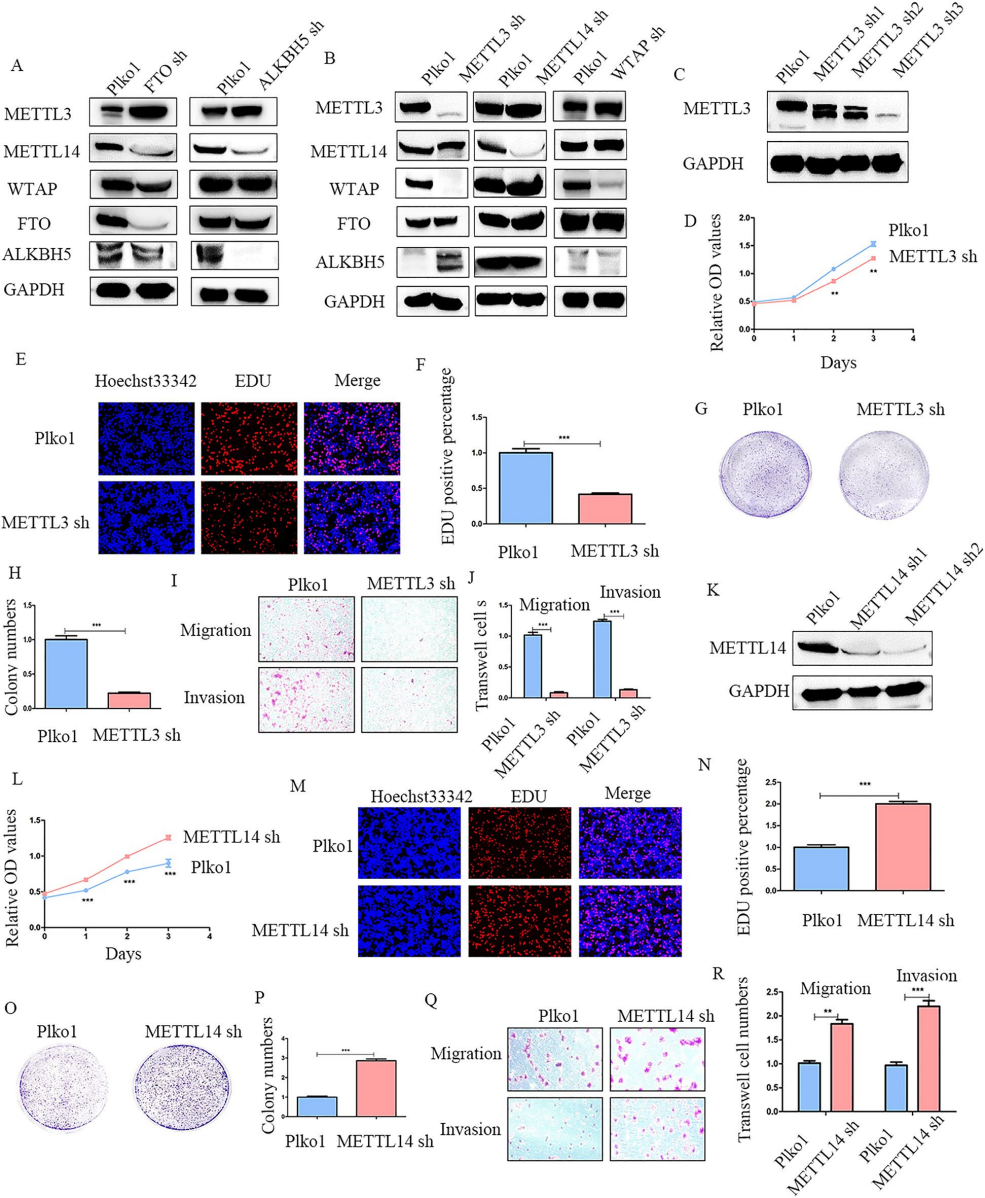
Silenced FTO/ALKBH5 promotes METTL3 while inhibits METTL14 to accelerating the development of CRC **A** The protein levels of METTL3, METTL14, and WTAP, when FTO and ALKBH5 were knockdown in HCT116 cells, showed by western blot. **B** Western blot indicated the expression of FTO and ALKBH5 in METTL3/METTL14/WTAP deficient cells. **C** The efficiency of METTL3 knockdown in CRC cells was verified by western blot. **D** Viability of METTL3 knockdown cells detected by CCK-8 assay **E**, **F** EdU analysis of the effects of METTL3 knockdown (magnification times, 200 ×). **G**, **H** Representative images of colony formation assay and colony number analysis. **I**, **J** Cell migration and invasion assays of CRC cells treated with METTL3 knockdown (magnification times, 100 ×). **K** The expression of METTL14 in METTL14 knockdown cells is indicated by western blot. **L**–**P** The results of CCK-8, colony formation, and EdU (magnification times, 200 ×) assays in METTL14 knockdown cells which be used to elevate cell proliferation. **Q**, **R** Migration and invasion were detected by transwell with METTL14 knockdown in CRC cells (magnification times, 100 ×). **p < 0.01; ***p < 0.001

We assumed whether FTO and ALKBH5 could influence other m6A regulators such as METTL3, METTL14, and WTAP expression. Therefore, the levels of m6A writers were detected in FTO/ ALKBH5 knockdown cells and it revealed that METTL3 was negatively correlated with FTO and ALKBH5, while METTL14 was positively correlated with FTO and ALKBH5 (Fig. 8A, B). Subsequently, we investigated the bio-function of METTL3 and METTL14 in CRC cells. Stable CRC cell lines with METTL3 and METTL14 knockdown were constructed and examined by western blot (Fig. 8C, K). CCK-8 assays demonstrated that METTL3 knockdown significantly decreased CRC cell proliferation (Fig. 8D). Meanwhile, both colony formation and EdU assays showed that METTL3 silencing inhibited the proliferation of CRC cells (Fig. 8E–H). Moreover, transwell assays indicated that METTL13 weakened the migration and invasion of CRC cells (Fig. 8I, J). We also knocked down METTL14 in CRC cells using shRNA. The results demonstrated that METTL14 silencing increased the malignant behaviors of CRC cells in proliferation (Fig. 8L–N), colony formation (Fig. 8O, P), migration, and invasion (Fig. 8Q, R). Furthermore, western blot indicated that down-regulation of METTL3 suppressed HK2 thus inhibiting FOXO pathway, while silence METTL14 promoted FOXO pathway through facilitation HK2 (Additional file 4: Figure S4G).

## Discussion

The genesis and development of tumors are closely related to the metabolic reprogramming of cancer cells, especially lipid metabolism and glucose metabolism [10, 17]. Studies have shown a dose–response relationship between body mass index (BMI) and CRC. For every 2 kg/m^2^ increase in BMI, CRC cancer risk increases by 4–10%, and for every 2 cm increase in waist circumference, CRC risk increases by 2–5% [18]. The nutritional availability of cancer cells in the tumor micro-environment constantly changes during tumor progression, whereby they can utilize abundant lipids to promote rapid cell growth [19, 20]. Studies have shown that m6A modification dynamically changes with the external environment [21, 22]. For instance, a high-fat environment can lead to changes in m6A-related molecules. This study demonstrated that m6A erasers (FTO and ALKBH5) were significantly down-regulated in CRC tissues compared with adjacent normal tissues. Moreover, obese CRC patients had lower levels of FTO and ALKBH5 compared with thinner CRC patients. Similarly, sodium palmitate reduced the expression of FTO and ALKBH5 in CRC cells. Cell function experiments indicated that both FTO and ALKBH5 had inhibitory effects on CRC cells. Furthermore, silencing FTO/ALKBH5 decreased METTL14, while increasing METTL3 levels. Importantly, METTL3 has been reported to act as an oncogene in CRC. Nevertheless, METTL14 played an anti-cancer role in CRC.

Previous studies have shown that FTO and ALKBH5 play a cancer-suppressive role in CRC. Wang et al. revealed that reduced FTO protein expression, whose ubiquitin-mediated protein degradation was increased in hypoxic conditions by the E3 ligase STRAP, was associated with a higher recurrence rate and poorer prognosis in patients with CRC. Mechanistically, FTO acts as a tumor suppressor by inhibiting MTA1 expression, recognized by the m6A reader IGF2BP2, in an m6A-dependent manner [23]. Zhang et al. demonstrated that lower ALKBH5 levels were closely associated with poorer prognosis in CRC patients. Functionally, down-regulation of ALKBH5 promoted cell proliferation, migration, and invasion, while up-regulation of ALKBH5 decreased the malignant behaviors of CRC cells. Mechanistically, MeRIP-seq and RNA-seq indicated that PHF20 was regulated by ALKBH5-mediated m6A modification by decreasing the stability of its mRNA in 3'UTR [24]. Chen et al. found that METTL3 promoted CRC cell proliferation by activating the m6A-GLUT1-mTORC1 axis and is a therapeutic target in CRC [12]. The following year, the same team reported that METTL3 inhibited anti-tumor immunity by targeting the m6A-BHLHE41-CXCL1/CXCR2 axis to promote CRC cell proliferation [25]. Wang et al. demonstrated that METTL14-mediated N6-methyladenosine modification of SOX4 mRNA inhibited tumor metastasis in CRC [26]. Yang et al. illustrated that METTL14 suppressed CRC proliferation and metastasis by down-regulating oncogenic lncRNA XIST [27]. Our results are consistent with the above studies, indicating that both FTO and ALKBH5 are down-regulated in CRC, hence, playing a role as tumor suppressors. The downstream gene is HK2, whereby FTO and ALKBH5 co-operatively negatively regulate HK2 expression in m6A-dependent manners. Moreover, FTO and ALKBH5 function as tumor suppressors by inhibiting METTL3 while promoting METTL14 expression.

HK2 is the first rate-limiting enzyme of the glycolytic pathway [28]. Warburg discovered the high glucose metabolism of tumors more than 70 years ago [29]. Approximately 60% of ATP in tumor cells comes from glycolysis. It has been confirmed that HK2 content is increased in the resected tissues of the lung, gastrointestinal, and breast malignant tumors. Additionally, it has been found that HK2 activity is higher when the lesions metastasize in breast cancer [30]. Hence, HK2 is essential for the metabolic behavior of rapidly growing tumors. At the gene level, the increase in HK2 expression was mainly due to the increase in its gene transcription. The promoter of HK2 has a wide range of signal transduction cascade activation pathways. Theoretically, the energy metabolism of tumors can be affected by the inhibition of glucose metabolism regulators, while normal cells remain unaffected [31, 32]. Therefore, targeting HK2 may provide a new strategy for the treatment of hyper-glucose-metabolized tumors.

## Conclusion

In conclusion, we observed the reduced FTO and ALKBH5 expression levels in CRC tissues and cell lines, whereby FTO and ALKBH5 were robustly down-regulated in obese CRC patients and CRC cells treated with sodium palmitate. In terms of their mechanism of action, down-regulation of FTO and ALKBH5 recognized HK2 m6A modification by IGF2BP2 promoted glucose metabolism via triggering the FOXO signaling pathway. Our results highlighted the crucial significance of m6A methylation in regulating CRC cell function, providing a promising therapeutic target of m6A modulators in CRC.

### Supplementary Information

**Additional file 1: Figure S1.** FTO over-expression inhibits proliferation, migration, and invasion of CRC (A) The FTO protein expression levels were detected by western blot. (B-E) CCK-8 and EdU (magnification times, 200 ×) results of FTO over-expression in HCT116 and SW620 cells. (F, G) Colony formation results of FTO over-expression in HCT116 and SW620 cells, together with its statistical chart. (H-J) Migration and invasion results of FTO over-expression in HCT116 cells and SW620 cells, together with its statistical chart (magnification times, 100 ×). **p < 0.01,***p < 0.001.

**Additional file 2: Figure S2.** Up-regulation of ALKBH5 hinders cell proliferation, migration, and invasion of CRC (A) Western blot analysis of transfection efficiency of over-expression ALKBH5 in HCT116 and SW620 cells. (B-G) The proliferation ability of ALKBH5 over-expression in HCT116 and SW620 cells was evaluated by CCK-8, colony formation, and EdU assays (magnification times, 200 ×). (H-J) The cell migration and invasion activities of HCT116 and SW620 cells with ALKBH5 over-expression were assessed by transwell assays (magnification times, 100 ×). **p < 0.01; ***p < 0.001.

**Additional file 3: Figure S3.** HK2 inhibitor 2DG restrains oncogenic effect induced by FTO/ALKBH5 silence (A) Western blot showing HK2 and FOXO1 protein levels in treated with FTO/ALKBH5 knockdown and 2DG in HCT116 cells. (B) CCK-8 indicated the ability of proliferation in FTO/ALKBH5 deficient CRC cells was rescued by 2DG treatment. (C-D) Colony formation was performed in FTO/ALKBH5 knockdown and 2DG treated HCT116 cells. (E–G) Analysis of cell migration and invasion while treated with 2DG in FTO/ALKBH5 deficient HCT116 cells (magnification times, 100 ×).*p < 0.05; **p < 0.01; ***p < 0.001.

**Additional file 4: Figure S4.** HK2 inhibitor 2DG decreases cell proliferation, migration and invasion in vitro (A) HK2 and FOXO1 protein levels were detected by western blot in HCT116 cells. (B-D) CCK8 and colony formation assay was performed to indicated effect of 2DG in proliferation in HCT116 cells. (E–F) Transwell assay showing 2DG’s effect on cell migration and invasion in HCT116 cells (magnification times, 100 ×). (G) Western blot indicated HK2 and FOXO1 protein level after METTL3 / METTL14 silence. ***p < 0.001.

**Additional file 5: Figure S5.** FTO and ALKBH5 inhibits glucose metabolism but does not affect cell apoptosis. RIP-QPCR showing HK2 m6A level in FTO/ALKBH5 knockdown or over-expression cells. (B) Glucose levels were detected in FTO/ALKBH5 knockdown or over-expression cells of HCT116. (C) ATP levels were mesured in FTO/ALKBH5 silence or up-regulation cells of HCT116. (D) Flow cytometry of FTO/ALKBH5 over-expression by FITC/PI staining. (E) Tunel staining was performed in tumor tissues of FTO/ALKBH5 over-expression. (F) Western blot assay was performed in FTO/ALKBH5 over-expression for BCL2 and Caspase3 proteins. **p < 0.01; ***p < 0.001.

**Additional file 6: Table S1.** Short hairpin targets. **Table S2.** Primers of genes. **Table S3.** Antibody information.

## Data Availability

All data generated or analyzed during this study are included in this published article.

## Abbreviations

ALKBH5: Alkylation repair homolog 5
CCK8: Cell counting kit-8
CRC: Colorectal cancer
DMEM: Dulbecco's modified eagle medium
EdU: 5-Ethynyl-2’-deoxyuridine
FBS: Fetal bovine serum
FTO: Fat-mass and obesity-associated protein
GAPDH: Glyceraldehyde-3-phosphate dehydrogenase
GO: Gene ontology
IHC: Immunohistochemistry
IGF2BP: Insulin-like growth factor 2 mRNA-binding proteins
KEGG: Kyoto Encyclopedia of Genes and Genomes
METTL3: Methyltransferase-like 3
METTL14: Methyltransferase-like 14
MeRIP-seq: Methylated RNA immunoprecipitation
m6A: N6-methyladenosine
PBS: Phosphate buffered saline
QPCR: Quantitative real-time polymerase chain reactio
RIP: RNA immunoprecipitation
RNA-seq: RNA-sequencing
shRNA: Short hairpin RNA
WTAP: Wilms tumor 1 associated protein

## References

[1] Yan H, Talty R, Aladelokun O, Bosenberg M, Johnson CH (2023). Ferroptosis in colorectal cancer: a future target?. Br J Cancer.

[2] Li W, Ke C, Yang C, Li J, Chen Q, Xia Z (2023). LncRNA DICER1-AS1 promotes colorectal cancer progression by activating the MAPK/ERK signaling pathway through sponging miR-650. Cancer Med.

[3] He J, Zhou M, Yin J, Wan J, Chu J, Jia J (2021). METTL3 restrains papillary thyroid cancer progression via m(6)A/c-Rel/IL-8-mediated neutrophil infiltration. Mol Ther.

[4] Jia J, Wu S, Jia Z, Wang C, Ju C, Sheng J (2022). Novel insights into m(6)A modification of coding and non-coding RNAs in tumor biology: from molecular mechanisms to therapeutic significance. Int J Biol Sci.

[5] Gao R, Ye M, Liu B, Wei M, Ma D, Dong K (2021). m6A modification: a double-edged sword in tumor development. Front Oncol.

[6] Wang C, Zhou M, Zhu P, Ju C, Sheng J, Du D (2022). IGF2BP2-induced circRUNX1 facilitates the growth and metastasis of esophageal squamous cell carcinoma through miR-449b-5p/FOXP3 axis. J Exp Clin Cancer Res.

[7] Zhang TP, Li R, Wang LJ, Li HM (2022). Impact of m6A demethylase (ALKBH5, FTO) genetic polymorphism and expression levels on the development of pulmonary tuberculosis. Front Cell Infect Microbiol.

[8] Xu A, Zhang J, Zuo L, Yan H, Chen L, Zhao F (2022). FTO promotes multiple myeloma progression by posttranscriptional activation of HSF1 in an m(6)A-YTHDF2-dependent manner. Mol Ther.

[9] Yadav P, Subbarayalu P, Medina D, Nirzhor S, Timilsina S, Rajamanickam S (2022). M6A RNA methylation regulates histone ubiquitination to support cancer growth and progression. Cancer Res.

[10] Liu C, Li C, Liu Y (2022). The role of metabolic reprogramming in pancreatic cancer chemoresistance. Front Pharmacol.

[11] Huang Y, Zhu C, Liu P, Ouyang F, Luo J, Lu C (2023). L1CAM promotes vasculogenic mimicry formation by miR-143-3p-induced expression of hexokinase 2 in glioma. Mol Oncol.

[12] Chen H, Gao S, Liu W, Wong CC, Wu J, Wu J (2021). RNA N(6)-Methyladenosine Methyltransferase METTL3 facilitates colorectal cancer by activating the m(6)A-GLUT1-mTORC1 axis and is a therapeutic target. Gastroenterology.

[13] Qiu X, Xu Q, Liao B, Hu S, Zhou Y, Zhang H (2022). Circ-CCS regulates oxaliplatin resistance via targeting miR-874-3p/HK2 axis in colorectal cancer. Histol Histopathol.

[14] Wang Z, Wang MM, Geng Y, Ye CY, Zang YS (2022). Membrane-associated RING-CH protein (MARCH8) is a novel glycolysis repressor targeted by miR-32 in colorectal cancer. J Transl Med.

[15] Li Y, He L, Wang Y, Tan Y, Zhang F (2022). N(6)-methyladenosine methyltransferase KIAA1429 elevates colorectal cancer aerobic glycolysis via HK2-dependent manner. Bioengineered.

[16] Zhang B, Chan SH, Liu XQ, Shi YY, Dong ZX, Shao XR (2021). Targeting hexokinase 2 increases the sensitivity of oxaliplatin by Twist1 in colorectal cancer. J Cell Mol Med.

[17] Zhu L, Zhu X, Wu Y (2022). Effects of glucose metabolism, lipid metabolism, and glutamine metabolism on tumor microenvironment and clinical implications. Biomolecules.

[18] Moghaddam AA, Woodward M, Huxley R (2007). Obesity and risk of colorectal cancer: a meta-analysis of 31 studies with 70,000 events. Cancer Epidemiol Biomarkers Prev.

[19] Huang H, Chen Y, Yin N, Li G, Ye S, Guo L (2023). Unsaturated fatty acid liposomes selectively regulate glutathione peroxidase 4 to exacerbate lipid peroxidation as an adaptable liposome platform for anti-tumor therapy. Mol Pharm.

[20] Kou Y, Geng F, Guo D (2022). Lipid metabolism in glioblastoma: from de novo synthesis to storage. Biomedicines.

[21] Phillips S, Mishra T, Khadka S, Bohan D, Espada CE, Maury W (2023). Epitranscriptomic N(6)-Methyladenosine profile of SARS-CoV-2-infected human lung epithelial cells. Microbiol Spectr.

[22] Liang J, Sun J, Zhang W, Wang X, Xu Y, Peng Y (2023). Novel insights into the roles of N(6)-methyladenosine (m(6)A) modification and autophagy in human diseases. Int J Biol Sci.

[23] Ruan DY, Li T, Wang YN, Meng Q, Li Y, Yu K (2021). FTO downregulation mediated by hypoxia facilitates colorectal cancer metastasis. Oncogene.

[24] Zhang Z, Wang L, Zhao L, Wang Q, Yang C, Zhang M (2022). N6-methyladenosine demethylase ALKBH5 suppresses colorectal cancer progression potentially by decreasing PHF20 mRNA methylation. Clin Transl Med.

[25] Chen H, Pan Y, Zhou Q, Liang C, Wong CC, Zhou Y (2022). METTL3 inhibits antitumor immunity by targeting m(6)A-BHLHE41-CXCL1/CXCR2 axis to promote colorectal cancer. Gastroenterology.

[26] Chen X, Xu M, Xu X, Zeng K, Liu X, Pan B (2020). METTL14-mediated N6-methyladenosine modification of SOX4 mRNA inhibits tumor metastasis in colorectal cancer. Mol Cancer.

[27] Yang X, Zhang S, He C, Xue P, Zhang L, He Z (2020). METTL14 suppresses proliferation and metastasis of colorectal cancer by down-regulating oncogenic long non-coding RNA XIST. Mol Cancer.

[28] Bao C, Zhu S, Song K, He C (2022). HK2: a potential regulator of osteoarthritis via glycolytic and non-glycolytic pathways. Cell Commun Signal.

[29] Liu J, van der Hoeven R, Kattan WE, Chang JT, Montufar-Solis D, Chen W (2023). Glycolysis regulates KRAS plasma membrane localization and function through defined glycosphingolipids. Nat Commun.

[30] Samec M, Liskova A, Koklesova L, Samuel SM, Zhai K, Buhrmann C (2020). Flavonoids against the Warburg phenotype-concepts of predictive, preventive and personalised medicine to cut the Gordian knot of cancer cell metabolism. EPMA J.

[31] Ciscato F, Ferrone L, Masgras I, Laquatra C, Rasola A (2021). Hexokinase 2 in cancer: a prima donna playing multiple characters. Int J Mol Sci.

[32] Rabbani N, Thornalley PJ (2019). Hexokinase-2 glycolytic overload in diabetes and ischemia-reperfusion injury. Trends Endocrinol Metab.

