# Supplementary figures and images for: Down-regulated FTO and ALKBH5 co-operatively activates FOXO signaling through m6A methylation modification in HK2 mRNA mediated by IGF2BP2 to enhance glycolysis in colorectal cancer
Source: Cell Biosci. 2023 Aug 14;13:148. doi: 10.1186/s13578-023-01100-9 (PMC10424385; doi:10.1186/s13578-023-01100-9)

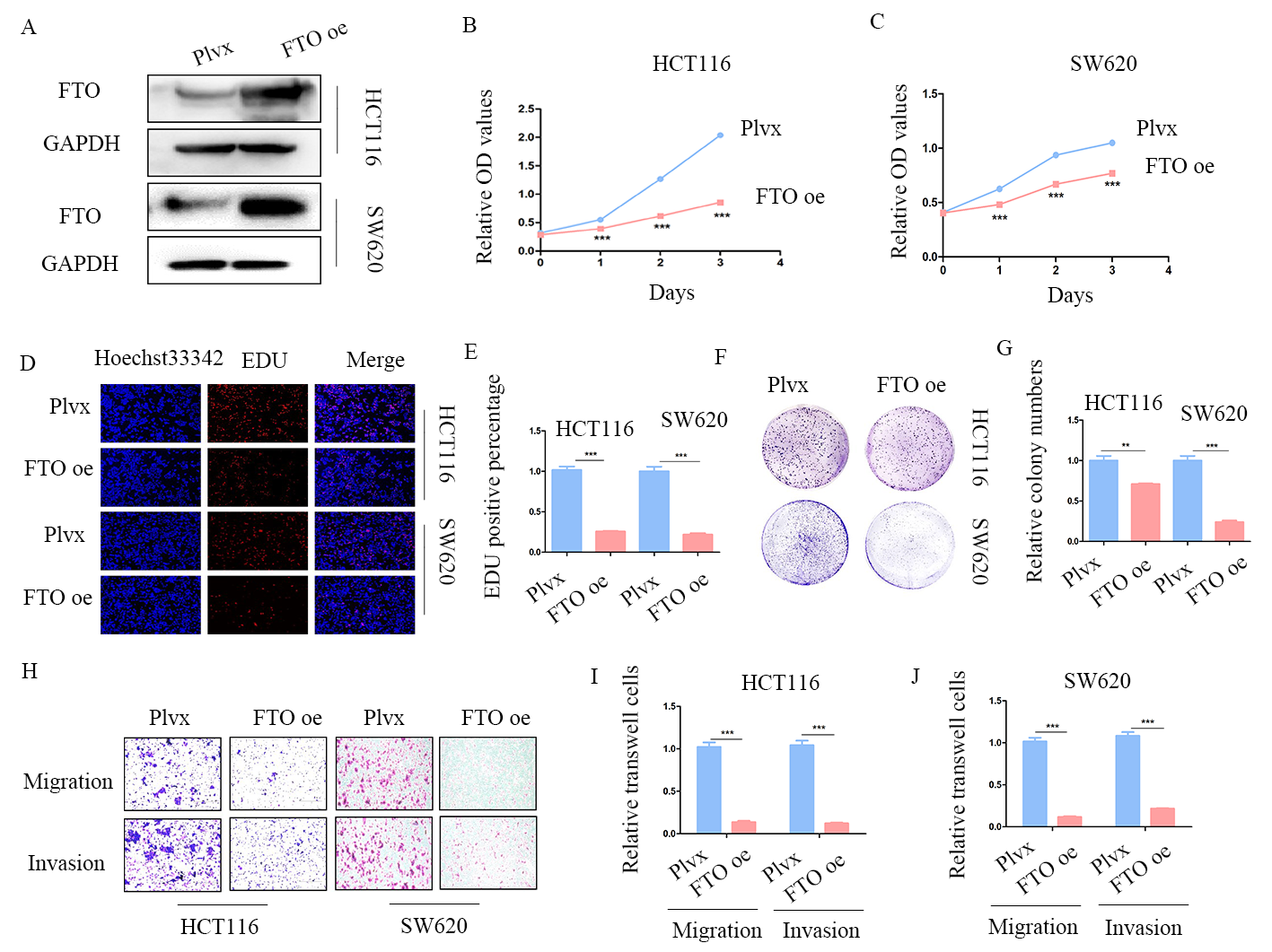

Supplement: Supplementary file 1 — Additional file 1: Figure S1. FTO over-expression inhibits proliferation, migration, and invasion of CRC (A) The FTO protein expression levels were detected by western blot. (B-E) CCK-8 and EdU (magnification times, 200 ×) results of FTO over-expression in HCT116 and SW620 cells. (F, G) Colony formation results of FTO over-expression in HCT116 and SW620 cells, together with its statistical chart. (H-J) Migration and invasion results of FTO over-expression in HCT116 cells and SW620 cells, together with its statistical chart (magnification times, 100 ×). **p < 0.01,***p < 0.001. [file 13578_2023_1100_MOESM1_ESM.tif]

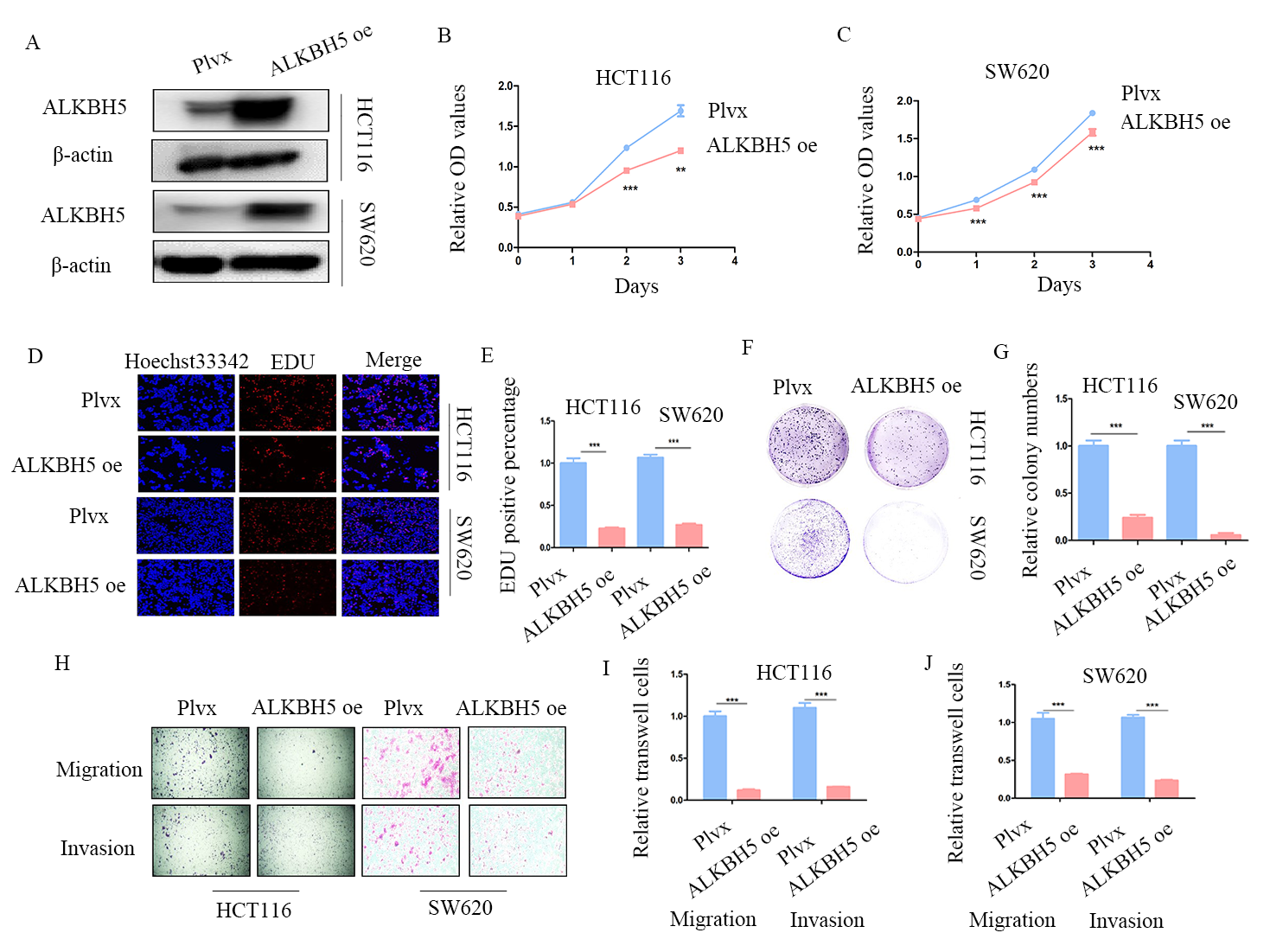

Supplement: Supplementary file 2 — Additional file 2: Figure S2. Up-regulation of ALKBH5 hinders cell proliferation, migration, and invasion of CRC (A) Western blot analysis of transfection efficiency of over-expression ALKBH5 in HCT116 and SW620 cells. (B-G) The proliferation ability of ALKBH5 over-expression in HCT116 and SW620 cells was evaluated by CCK-8, colony formation, and EdU assays (magnification times, 200 ×). (H-J) The cell migration and invasion activities of HCT116 and SW620 cells with ALKBH5 over-expression were assessed by transwell assays (magnification times, 100 ×). **p < 0.01; ***p < 0.001. [file 13578_2023_1100_MOESM2_ESM.tif]

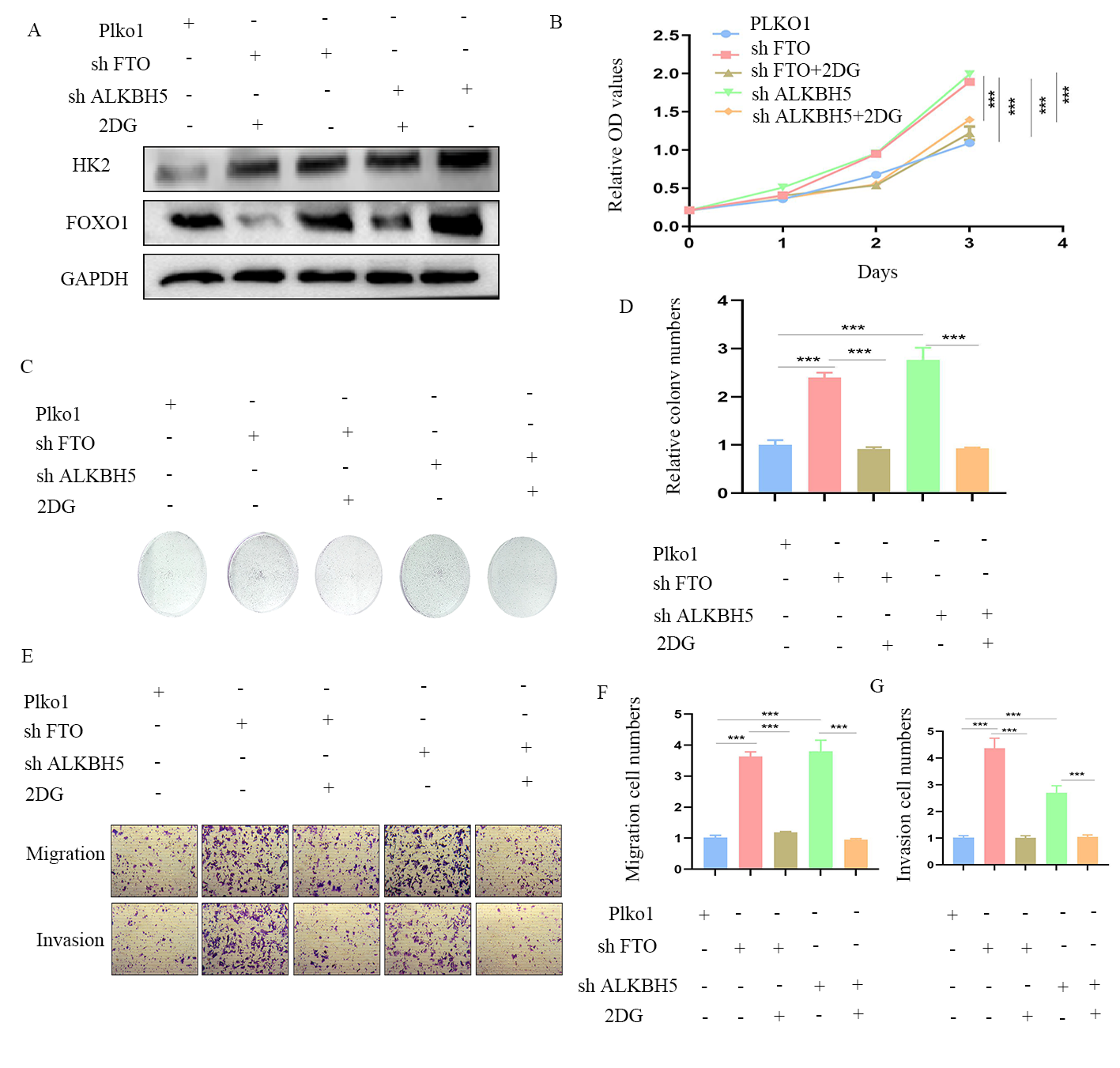

Supplement: Supplementary file 3 — Additional file 3: Figure S3. HK2 inhibitor 2DG restrains oncogenic effect induced by FTO/ALKBH5 silence (A) Western blot showing HK2 and FOXO1 protein levels in treated with FTO/ALKBH5 knockdown and 2DG in HCT116 cells. (B) CCK-8 indicated the ability of proliferation in FTO/ALKBH5 deficient CRC cells was rescued by 2DG treatment. (C-D) Colony formation was performed in FTO/ALKBH5 knockdown and 2DG treated HCT116 cells. (E–G) Analysis of cell migration and invasion while treated with 2DG in FTO/ALKBH5 deficient HCT116 cells (magnification times, 100 ×).*p < 0.05; **p < 0.01; ***p < 0.001. [file 13578_2023_1100_MOESM3_ESM.tif]

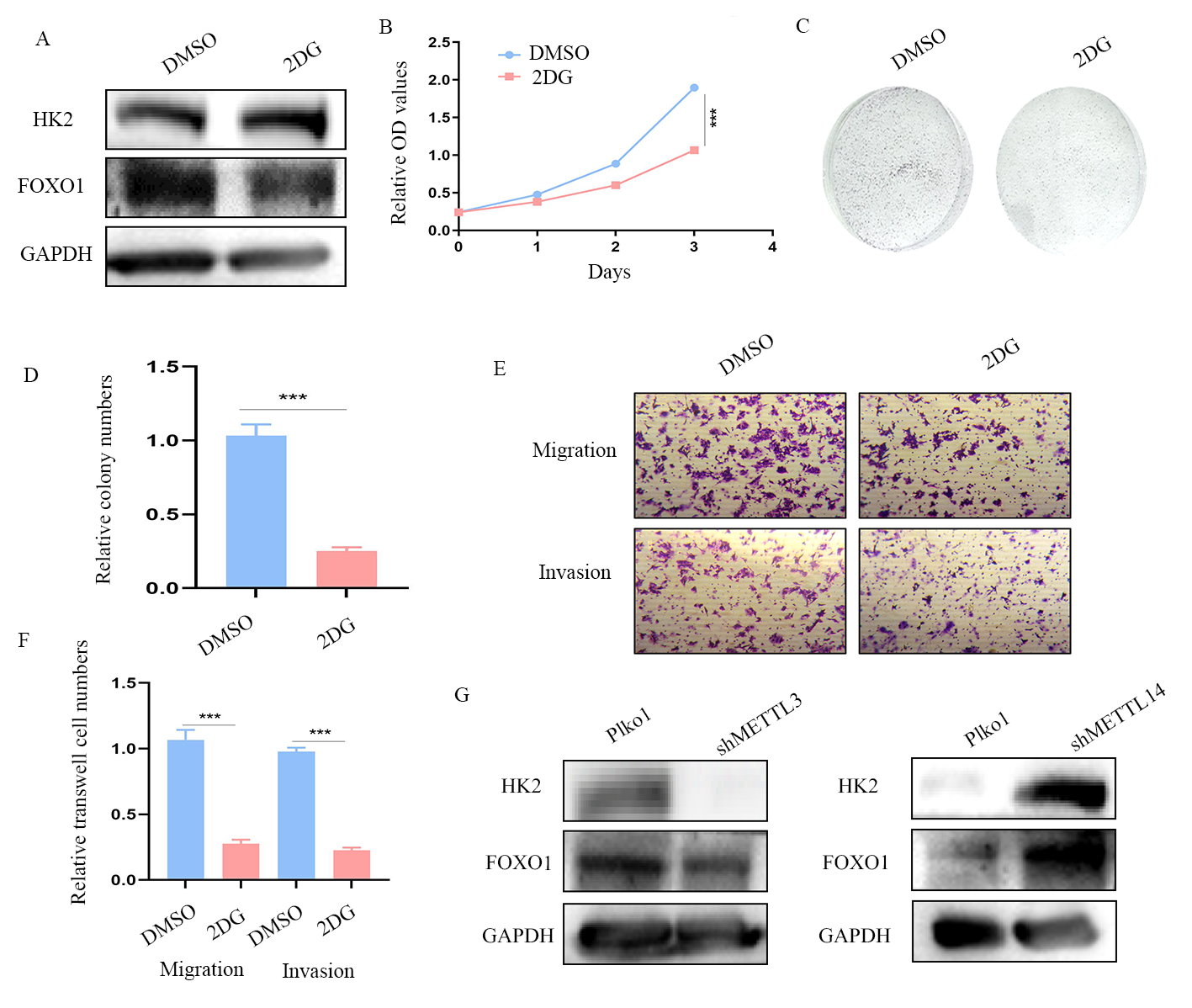

Supplement: Supplementary file 4 — Additional file 4: Figure S4. HK2 inhibitor 2DG decreases cell proliferation, migration and invasion in vitro (A) HK2 and FOXO1 protein levels were detected by western blot in HCT116 cells. (B-D) CCK8 and colony formation assay was performed to indicated effect of 2DG in proliferation in HCT116 cells. (E–F) Transwell assay showing 2DG’s effect on cell migration and invasion in HCT116 cells (magnification times, 100 ×). (G) Western blot indicated HK2 and FOXO1 protein level after METTL3 / METTL14 silence. ***p < 0.001. [file 13578_2023_1100_MOESM4_ESM.tif]

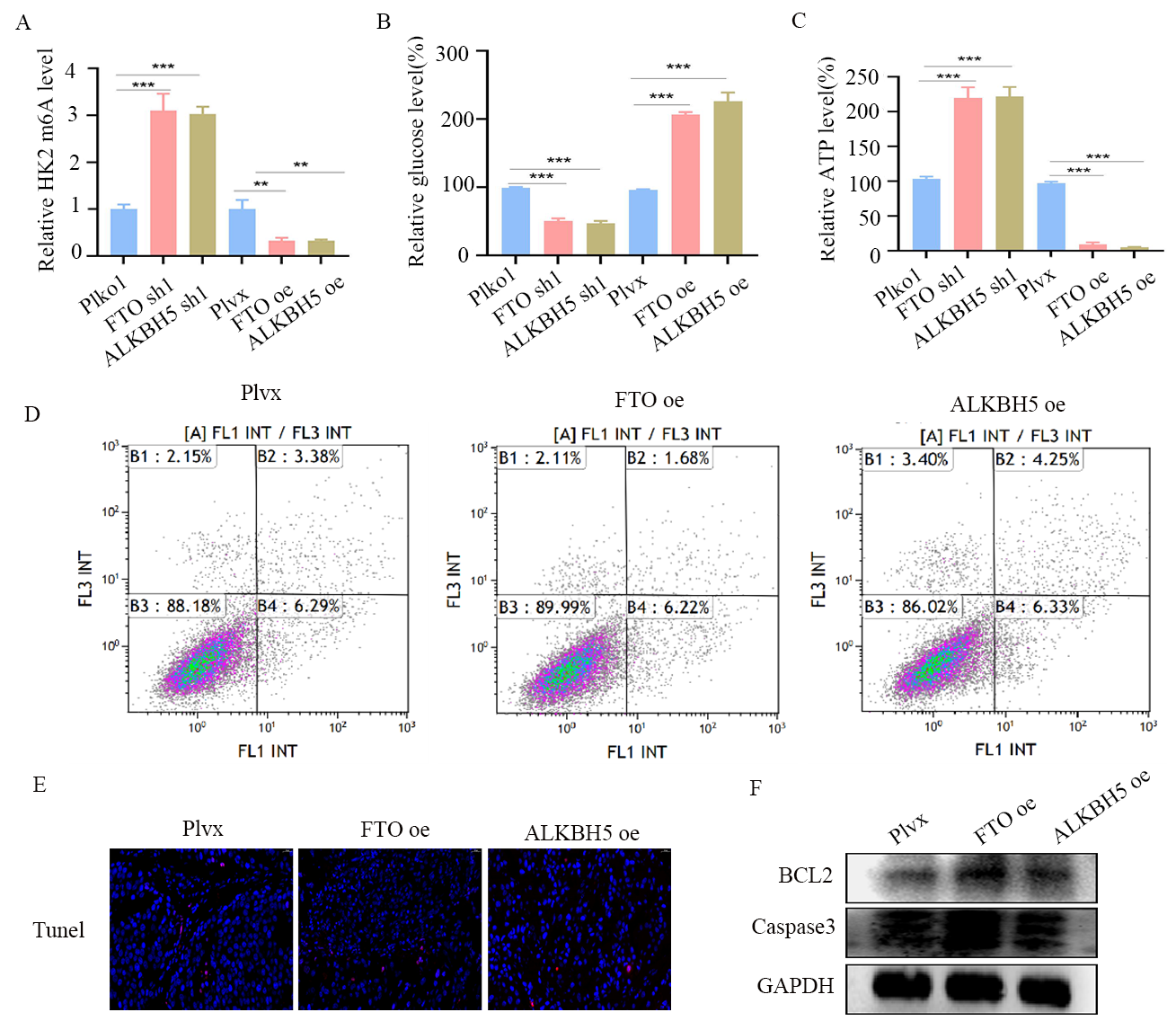

Supplement: Supplementary file 5 — Additional file 5: Figure S5. FTO and ALKBH5 inhibits glucose metabolism but does not affect cell apoptosis. RIP-QPCR showing HK2 m6A level in FTO/ALKBH5 knockdown or over-expression cells. (B) Glucose levels were detected in FTO/ALKBH5 knockdown or over-expression cells of HCT116. (C) ATP levels were mesured in FTO/ALKBH5 silence or up-regulation cells of HCT116. (D) Flow cytometry of FTO/ALKBH5 over-expression by FITC/PI staining. (E) Tunel staining was performed in tumor tissues of FTO/ALKBH5 over-expression. (F) Western blot assay was performed in FTO/ALKBH5 over-expression for BCL2 and Caspase3 proteins. **p < 0.01; ***p < 0.001. [file 13578_2023_1100_MOESM5_ESM.tif]
