# Supplementary material for: Down-regulated FTO and ALKBH5 co-operatively activates FOXO signaling through m6A methylation modification in HK2 mRNA mediated by IGF2BP2 to enhance glycolysis in colorectal cancer
Source: Cell Biosci. 2023 Aug 14;13:148. doi: 10.1186/s13578-023-01100-9 (PMC10424385; doi:10.1186/s13578-023-01100-9)
Supplement: Supplementary file 6 — Additional file 6: Table S1. Short hairpin targets. Table S2. Primers of genes. Table S3. Antibody information. [file 13578_2023_1100_MOESM6_ESM.docx]

**Supplementary Table1 Short hairpin targets**

| **Gene names** | **Target sequence(5’-3’)** |
| --- | --- |
| IGF2BP1 sh1 | GCAGTGGTGAATGTCACCTAT |
| IGF2BP1 sh2 | CTCCAAAGTTCGTATGGTTAT |
| IGF2BP2 sh1 | AGTGAAGCTGGAAGCGCATAT |
| IGF2BP2 sh2 | CAGTGCTGAGATAGAGATTAT |
| IGF2BP3 sh1 | GAAACTTCAGATACGAAATAT |
| IGF2BP3 sh2 | TCTGCGGCTTGTAAGTCTATT |
| HK2 | GCCTGGCTAACTTCATGGATA |
| METTL3 sh1 | GCCAAGGAACAATCCATTGTT |
| METTL3 sh2 | GCAAGTATGTTCACTATGAAA |
| METTL3 sh3 | CGTCAGTATCTTGGGCAAGTT |
| METTL14 sh1 | GAACCTGAAATTGGCAATATA |
| METTL14 sh2 | GAAGACGCCTTCATCTATTTG |
| WTAP sh | GGCAAGTACACAGATCTTAAC |
| FTO sh1 | TCACCAAGGAGACTGCTATTT |
| FTO sh2 | CGGTTCACAACCTCGGTTTAG |
| FTO sh3 | ACCTGAACACCAGGCTCTTTA |
| ALKBH5 sh1 | CCACCCAGCTATGCTTCAGAT |
| ALKBH5 sh2 | GAAAGGCTGTTGGCATCAATA |
| ALKBH5 sh3 | CCTCAGGAAGACAAGATTAGA |

**Supplementary Table2 Primers of genes**

| **Gene names** | **Sequence(5’-3’)** |
| --- | --- |
| HK2 forward | GAGCCACCACTCACCCTACT |
| HK2 reverse | CCAGGCATTCGGCAATGTG |
| FTO forward | GCTGCTTATTTCGGGACCTG |
| FTO reverse | AGCCTGGATTACCAATGAGGA |
| ALKBH5 forward | CGGCGAAGGCTACACTTACG |
| ALKBH5 reverse | CCACCAGCTTTTGGATCACCA |
| GAPDH forward | GGAGCGAGATCCCTCCAAAAT |
| GAPDH reverse | GGCTGTTGTCATACTTCTCATGG |

**SupplementaryTable3 Antibody information**

| **Antibody** | **Company** | **Catalogue** | **Dilution ratio** |
| --- | --- | --- | --- |
| GAPDH | Proteintech | 60004-1-Ig | 1：5000 |
| β-Tubulin | Proteintech | 10068-1-AP | 1：5000 |
| Ki67 | Proteintech | 27309-1-AP | 1：2000 |
| METTL3 | Abcam | ab195352 | 1：1000 |
| METTL14 | Abcam | ab300104 | 1：1000 |
| WTAP | Abcam | ab195380 | 1：1000 |
| β-Actin | Proteintech | 60008-1-Ig | 1：5000 |
| HK2 | Abcam | ab209847 | 1：1000 |
| FTO | Abcam | ab126605 | 1：1000 |
| ALKBH5 | Abcam | ab195377 | 1：1000 |
| FOXO1 | CST | 2880S | 1：1000 |
| Goat Anti-Mouse IgG | CWBIO | CW0102S | 1：5000 |
| Goat Anti-Rabbit IgG | CWBIO | CW0103S | 1：2000 |
